# Pituitary and COVID-19 vaccination: a systematic review

**DOI:** 10.1007/s11102-024-01402-2

**Published:** 2024-05-18

**Authors:** Martina Verrienti, Valentino Marino Picciola, Maria Rosaria Ambrosio, Maria Chiara Zatelli

**Affiliations:** 1 Unit of Endocrinology and Metabolic Diseases, Department of Specialty Medicines, University Hospital of Ferrara, Ferrara, Italy; 2 Section of Endocrinology, Geriatrics and Internal Medicine, Department of Medical Sciences, University of Ferrara, Via Ariosto 35, Ferrara, 44100 Italy

**Keywords:** COVID-19, Vaccine, Pituitary, Hypophysitis, Pituitary apoplexy

## Abstract

**Purpose:**

This systematic review aims to examine the latest research findings and assess the impact of COVID-19 vaccination on the pituitary gland.

**Method:**

PubMed and Tripdatabase were searched from January 1st, 2020 to February 12th, 2024. Case reports, case series and reviews related to post COVID-19 vaccination pituitary disease were included. Eligible articles were tabulated and analysed in the attempt to provide an overview on the epidemiology, clinical presentation, imaging, treatment, outcomes and pathophysiological background of post COVID-19 vaccination pituitary disease.

**Results:**

Among the 23 case reports included in this review, post COVID-19 vaccination hypophysitis was reported in 9 patients, pituitary apoplexy (PA) in 6 cases, SIADH in 5 cases and Isolated ACTH deficiency in 2 cases. Additionally, precipitating adrenal crisis was registered in 7 patients and pituitary tumor enlargement in 1 patient after receiving COVID-19 vaccination.

**Conclusion:**

Despite the rarity of these events, our research findings suggest an association between COVID-19 vaccination and the subsequent development of pituitary diseases. The most common manifestations include hypophysitis with ADH deficiency, PA and SIADH, with symptoms typically emerging shortly after vaccine administration. Potential pathogenetic mechanisms include molecular mimicry, vaccine adjuvants and vaccine-induced thrombotic thrombocytopenia (VITT), with the presence of ACE2 receptors in the hypothalamus-pituitary system contributing to the process. These findings can aid in diagnostic and treatment decisions for patients presenting with these syndromes. Nevertheless, given the rarity of these events, safety and efficacy of the currently available COVID-19 vaccines remain robust and we strongly advocate continuing pursuing vaccination efforts.

## Introduction

Coronavirus disease 2019 (COVID-19) outbroke in late December 2019 in China, reaching pandemic proportions by March 2020. The causative agent, identified as SARS-CoV-2, is a novel betacoronavirus capable of causing from mild to severe pulmonary distress, with approximately 7 million deaths reported by January 2024 [1, 2]. The virus affects various systems, including the endocrine system via the Angiotensin converting enzyme 2 (ACE2) receptor [3].

In response to this widespread health crisis, a massive vaccination campaign since late 2020 has administered over 13 billion doses globally. The employed vaccines are based on different technologies targeting the virus’s spike glycoprotein [2], including nucleic acid (RNA, DNA), adenoviral vector, protein-based (subunit particle), and attenuated or weakened SARS-CoV‐2 virus vaccines (Table 1) [4]. The vaccination campaign prevented severe illness and hospitalization thereby alleviating healthcare burden. Common adverse events include flu-like symptoms, headache, local and allergic reactions [5]. Moreover, new-onset autoimmune diseases after COVID-19 vaccination have been reported, including autoimmune liver diseases, Guillain-Barré syndrome, rheumatoid arthritis, and systemic lupus erythematosus [6]. Vaccine-induced autoimmunity can be due to several mechanisms such as molecular mimicry, vaccine adjuvants that burst inflammatory responses, and adaptive immune response stimulation leading to hyperinflammatory conditions [6]. Vaccine-induced thrombotic thrombocytopenia (VITT) has also been described in healthy individuals following COVID-19 vaccination and it is attributed to platelet factor 4 (PF4) antibody-mediated platelet and complement activation [6].

**Table 1:** Overview of most used available vaccines

Drug company	AstraZeneca*	Pfizer-BioNTech*	Moderna*	Janssen (Jonson & Jonson)	Novovax
**Trade name**	Vaxzevria	Comirnaty	Spikevax	Jcovden	Nuvaxovid
**Type**	Viral vector	mRNA	mRNA	Viral vector	Spike protein

*Vaccines quoted in our Review

The endocrine consequences of COVID-19 vaccination are generally rare and benign. Thyroid alterations are the most common (subacute thyroiditis, Graves’ disease), followed by type 1 diabetes mellitus and transient changes in menstrual cycle length; the cases of adrenal hemorrhage are extremely rare, but more severe [7]. In addition, post-vaccination pituitary gland involvement has been reported. Assessing the impact of COVID-19 vaccines on pituitary gland is useful to better understand and, potentially, quantify the risk of post-vaccination pituitary disease. Therefore, in this systematic review we aim to examine the latest research findings concerning the impact of COVID-19 vaccines on the pituitary gland.

## Methods

We conducted a systematic literature review using PubMed (pubmed.ncbi.nlm.nih.gov) and Tripdatabase (tripdabase.it). Our search included the following terms: “COVID19 vaccin* OR SARS-CoV-2 vaccin*” in combination with “Pituitary”, “Hypophysitis”, “Diabetes insipidus”, “ADH deficiency”, “Pituitary apoplexy”, “Syndrome of inappropriate antidiuretic hormone secretion”, “Adrenal insufficiency”, “Hypopituitarism” in articles published from January 1st, 2020 to February 12th, 2024. Initially, we comprised articles referring to clinical studies, case series, case reports, posters, and abstracts, written in English.

No age limit was set to the study selection; therefore, all ages have been included in the search for source data. Existing reviews were also consulted to identify additional relevant articles. Articles were initially screened evaluating title and abstract, then examining full-text, including only those related to post COVID-19 vaccination pituitary disease. Additionally, we reviewed the reference list of the selected studies to identify any undetected report. Selected articles were tabulated, documenting patient demographics, vaccine details, symptoms presentation and onset, imaging findings, diagnosis, clinical management, and outcome.

## Results

Pituitary disorders following COVID-19 vaccination have been documented in 23 case reports, which are summarized in Table 2. These primarily encompass cases of pituitary apoplexy (PA) and hypophysitis, alongside other dysfunctions such as Syndrome of Inappropriate Antidiuretic Hormone Secretion (SIADH) and isolated adrenocorticotroph hormone (ACTH) deficiency (IAD). In the subsequent paragraphs, we provide an accurate review covering epidemiology, clinical presentation, imaging findings, treatments, outcomes, and potential pathogenesis of the primary pituitary diseases occurring post COVID-19 vaccination.

**Table 2:** Review of the literature

Studies	Patients		Vaccine			Clinical features					
*Reference*	*Age, Gender*	*Medical history*	*Type*	*Dose Number*	*Previous dose*	*Onset of Symptom*	*Presenting Symptoms*	*Imaging findings*	*New Hormonal Deficiency*	*Clinical Management*	*Outcome*
• **Hypophysitis**											
Murvelashvili & Tessnow, 2021	51, M	Healthy	Moderna	Nausea, malaise, and arthralgias 3 days after the first dose. 2 days after the second dose worsening nausea, vomiting, mid-epigastric abdominal pain.				MRI: asymmetric enlargement of the pituitary gland, pituitary stalk thickening	Secondary adrenal insufficiency, central hypothyroidism and hypogonadism	High-dose steroids and thyroid hormone replacement	MRI follow-up at 1 month: reduced enlargement of the pituitary gland with almost empty sella. Central hypogonadism recovered
Ankireddypalli et al., 2022	48, F	Obesity	Pfizer-BioNTech	Flu-like symptoms, severe headache, myalgia, polyuria and polydipsia 2 days after the first dose. After second dose worsening of polydipsia, polyuria, headache, and development of amenorrhea and lethargy.				MRI: pituitary stalk thickening, partially empty sella	Low IGF-1 level; low gonadotropin level; ADH deficiency	Nasal spray DDAVP 10 mcg twice daily	Persistence of ADH deficiency and pituitary stalk thickening during follow-up. Improvement of IGF1 levels and amenorrhea at 5 months evaluation
Bouca et al., 2022	37, F	Rheumatoid arthritis treated with adalimumab	Pfizer-BioNTech	2nd	Pfizer-BioNTech	7 days	Thirst, polyuria	MRI: posterior lobe high-intensity signal loss on T1	ADH deficiency	Oral DDAVP 0.06 mg twice a day	Persistent need of DDAVP during follow-up
Ach et al., 2022	54, F	Arterial hypertension	AstraZeneca	1st	-	3 day	Polyuria, nycturia, fatigue	MRI: pituitary stalk thickening	ADH deficiency	Oral DDAVP titrated to 120 mg/day	NA
Matsuo et al., 2023	74, F	Stable myelodysplastic syndrome	Moderna	4th	Pfizer-BioNTech	1 month	Thirst, polydipsia, polyuria and bilateral optic neuritis	MRI: pituitary gland enlargement, pituitary stalk thickening, posterior lobe high-intensity signal loss on T1, homogeneous enhancement of the whole pituitary gland and stalk after gadolinium	ADH deficiency	Nasal spray DDAVP 5 µg twice daily switched to oral DDAVP 240 µg twice daily	Persistence of ADH deficiency during follow-up
Ishay & Shacham, 2023	59, F	Crohn’s disease treated with mesalazine	Pfizer-BioNTech	1st	-	8 weeks	Thirst, polyuria, fatigue, weight loss	MRI: pituitary stalk thickening, posterior lobe high-intensity signal loss on T1	ADH deficiency	Oral DDAVP 0.2 mg twice daily	Persistence of ADH deficiency 18 months after vaccination
Partenope et al., 2023	16, M	Healthy	Symptoms onset few days after first dose of Pfizer-BioNTech vaccine and then worsened after the second dose				Polyuria, polydipsia, weight loss over the last 4 months	MRI: pituitary stalk thickening, posterior lobe high-intensity signal loss on T1	ADH deficiency	Oral DDAVP 60 mcg twice daily	Persistence of ADH deficiency after three months
Yu et al., 2023	59, M	Space-occupying lesion of unknown nature in sella turcica area diagnosed 30 years before	NA	2nd	NA	26 days	3 day anorexia after the first dose. 26 days after the second dose decreased appetite, vision loss, nausea, vomiting	MRI: cystic lesion within the sella, indicative of Rathke’s cleft cyst	Secondary hypocortisolism and hypothyroidism, GH deficiency, low LH and FSH with normal testosterone	Oral prednisone 5 mg/day and levothyroxine sodium tablets 50 ug/day	The patient developed hypogonadism 1 year after, resulting in anterior panhypopituitarism
Mazzeo et al., 2024	21, F	Hashimoto’s thyroiditis, obesity, impaired glucose tolerance	Pfizer-BioNTech	2nd	Pfizer-BioNTech	7	Polyuria and polydipsia	MRI: diffuse gland enlargement, pituitary stalk thickening, posterior lobe high-intensity signal loss on T1	ADH deficiency	Oral DDAVP 60 mg/day	Complete withdrawal of DDAVP and full and spontaneous recovery from ADH deficiency
• **Pituitary apoplexy**											
Jaggi & Jabbour, 2021	44, M	History of hypogonadism	NA	2nd	NA	3 days	Fever, chills, blurry vision, change in mental status, hypotension	MRI: sellar and suprasellar mass with optic chiasm compression and left sphenoidal extension	NA	Stress dose steroids, TSS	Patient was on maintenance dose steroids and levothyroxine
Aliberti et al., 2022	50, M	Kidney stones 15 years before	Moderna	3rd	Pfizer-BioNTech	1 day	Headache resistant to analgesics, vomit, nausea, diplopia, hypotension, fever	MRI: recent signs of haemorrhage in a macroadenoma, compressing the optic chiasm and extending to suprasellar cisterns and left cavernous sinus. Erosion of the floor and the back of the sella turcica. Pituitary stalk not visible.	Secondary hypogonadism; suspected central hypocortisolism	Dexamethasone 8 mg/ day; Morphine; TSS	At 4 months follow-up no residual pituitary adenoma, no abnormal pituitary hormones, no headache and diplopia
Piañar-Gutiérrez et al., 2022	37, F	NA	AstraZeneca	NA	NA	5 days	High-intensity frontal headache partially relieved by analgesics	MRI: signs of pituitary bleeding with a possible microadenoma	None	No treatment	Symptoms resolved within 2–3 weeks without any associated hormonal defects
Zainordin et al., 2022	24, F	History of headache since the age of 15, overweight	AstraZeneca	2nd	AstraZeneca	1 day	Severe frontal headache	MRI: pituitary gland heterogenous enhancement, extending to the suprasellar region. Pituitary stalk not visible, optic chiasm compression; cavernous sinus invasion	Suspected central hypocortisolism	High doses analgesia; hydrocortisone	Headache persistence required new hospitalization, suggesting hypophysitis. Hydrocortisone was shifted to dexamethasone, with beneficial outcome.
Roncati & Manenti, 2022	28, F	Healthy	AstraZeneca	38 °C fever for 24 h and tension-type headache for a full month after the first dose with intensification after the second dose. Developement of amenorrhea and hyperprolactinemia after the second dose.				MRI: pituitary hemorrhage	NA	NA	Menstrual recovery and MRI improvement at 3 months follow-up
Tanaka et al., 2023	45, M	Giant pituitary tumor with partial hypopituitarism and elevated PRL awaiting TSS	Pfizer-BioNTech	2nd	Pfizer-BioNTech	3 days	Progressively worsening headache, vomiting and decreased visual acuity	CT: hyperdense suprasellar mass lesion in the upper part of the pituitary tumor	NA	Stress dose hydrocortisone; TSS	Improvement of headache and visual acuity after surgery. Development of postoperative ADH deficiency. At 5 months MRI showed tumor shrinkage and decompression of the optic apparatus
• **SIADH**											
Lindner & Ryser, 2021	79, M	Ischemic cerebrovascular accident, Gastritis, GERD	Moderna	2nd	Moderna	9 days	Weakness, fatigue and anorexia.	NA	Hyponatremia, low serum osmolality, High sodium urine output	Fluid restriction (< 1000 ml/day), oral urea 30 g/day and then 45 g/day	After 10 days, sodium yielded a normal value and urea therapy was paused to assess SIADH recovery
Chienwichai et al., 2022	24, M	Use of kratom extract daily	AstraZeneca	2nd	Sinovac Biotech	1 day	High fever, severe headache, nausea, vomiting, lethargy.	CT: diffusely decreased brain parenchyma attenuation with brain oedema	Hyponatremia, low serum osmolality, High sodium urine output	Hypertonic saline 3% infusion until sodium level > 120 mEq/L and then fluid restriction (< 800 mL/day).	The patient was discharged 1 week after admission with no neurological defects or medications
Kumagai et al., 2023	84, F	Hypertension, atrial fibrillation	Pfizer-BioNTech	2nd	Pfizer-BioNTech	2 days	Nausea, vomiting, headache. Lethargy and impaired consciousness	NA	Hyponatremia, low serum osmolality, High sodium urine output	Intravenous saline infusion until symptoms resolution and plasma sodium level increase	Fluid therapy stop on day 4. The patient was discharged without symptoms
Dharma et al., 2023	48, F	Healthy	Pfizer-BioNTech	2nd	Pfizer-BioNTech	1 day	Severe headache and depressed consciousness. Urinary retention.	CT: cerebral oedema	Hyponatremia, low serum osmolality, inappropriately high copeptin levels	Empirical treatment for meningoencephalitis. Intravenous saline infusion switched to dextrose 5% infusion because of polyuria onset	Neurological symptoms resolution and sodium normalization in 13 h.
Yang et al., 2023	83, M	Chronic pancreatitis, COBP, transient ischemic attack	Pfizer-BioNTech	1st	-	3 days	Severe headache, generalized weakness and epilepsy development	MRI: mild brain atrophy	Hyponatremia, low serum osmolality, High sodium urine output	Hypertonic saline 3% infusion. Anti-epileptic drugs.	Crisis resolution after anti-epileptic treatment and sodium stabilization.
• **Isolated adrenal deficiency (IAD)**											
Morita et al., 2022	31, M	Healthy	Pfizer-BioNTech	2nd	Pfizer-BioNTech	1 day	General fatigue and fever for 7 days, followed by headache, nausea, diarrhea	MRI: moderate pituitary atrophy; preservation of high intensity of the posterior pituitary gland in T1	Isolated ACTH deficiency	Stress dose hydrocortisone followed by replacement therapy	MRI performed at one month after symptoms’ onset confirmed pituitary atrophy
Mizuno et al., 2022	48, M	Valproate and levetiracetam treated epilepsy, glomerulonephritis previously treated with steroids	Pfizer-BioNTech	1st	-	1 day	Fatigue, loss of appetite, fever, generalized weakness, altered mental status	MRI: no hypotalamic and pituitary abnormalities	Isolated ACTH deficiency	Stress dose hydrocortisone followed by replacement therapy	NA
• **Pituitary enlargement**											
Srimanan & Panyakorn, 2023	60, F	Allergic asthma and rhinitis	Pfizer-BioNTech	4th	Pfizer-BioNTech	3 days	Loss of vision	MRI: pituitary mass with suprasellar and sphenoidal extension, optic chiasm compression	Mild hyperprolactinemia and partial hypopituitarism	TSS	Six months after surgery no residual tumor detected at follow-up MRI.

ADH: anti diuretic hormone; CT: Computed Tomography; COBP: chronic obstructive bronchopneumopathy; DDAVP: Desmopressin; IAD: Isolated Adrenal Deficiency; MRI: Magnetic Resonance Imaging; SIADH: syndrome of inappropriate antidiuretic hormone

### Hypophysitis

#### Epidemiology

Post COVID-19 vaccination hypophysitis was reported in 9 patients, with a female-to-male ratio of 2:1. The average age of the affected individuals was 46.5 years (ranging from 16 to 74), including one previously healthy adolescent [8]. The majority of patients (88%) received a mRNA vaccine, with Pfizer/BioNTech (63%) and Moderna being the most common. Only one patient received the viral vector AstraZeneca vaccine. Notably, hypophysitis occurred regardless of the vaccine dose number, and only one clinical report noted testing negative for SARS-CoV-2 via nasopharyngeal swab [8]. Concerning patients’ clinical history, three female patients presented with obesity [9–11], three with autoimmunity, being affected respectively with Crohn’s disease, rheumatoid arthritis and Hashimoto’s thyroiditis and one female patient had stable myelodysplastic syndrome [11–14]. Additionally, one patient had a previously identified space-occupying sellar lesion [15].

#### Clinical presentation

All patients experienced symptoms, with a minority developing mild symptoms shortly after the first vaccination, which worsened following the second dose [8, 9, 16]. Most of the patients (78%) sought medical attention due to symptoms acute onset associated with vasopressin (AVP) deficit, presenting with polyuria, intense thirst and polydipsia [8–14]. A smaller proportion of patients reported associated weight loss [8, 12] and headache [9]. Laboratory evaluation typically revealed elevated serum and low urine osmolality [9, 10, 12, 14], with 43% of them exhibiting hypernatremia [10, 12, 14]. Notably, one patient, in addition to ADH deficiency, experienced transient amenorrhea with low gonadotropins and IGF-1 levels, which recovered 5 months post-vaccination [9]. In two male patients, hypophysitis manifested with anterior pituitary involvement [15, 16]. One patient displayed symptoms including nausea, vomiting, mid-epigastric pain, fatigue, and hyponatremia, with laboratory findings confirming severe, secondary adrenal insufficiency, central hypothyroidism, and hypogonadism [16]. Similarly, the other patient presented with symptoms and laboratory results consistent with adrenal insufficiency, central hypothyroidism and growth hormone deficiency [15]. Lastly, a case report presented by Matsuo et al. documented hypophysitis associated with optic neuritis [14].

#### Imaging findings

Magnetic resonance imaging (MRI) displayed pituitary stalk thickening in 70% of the patients [8–12, 14, 16], while the absence of high intensity (bright) signal in the posterior pituitary on T1 weighted imaging was described in 5 patients, all showing ADH deficiency [8, 11–14]. Pituitary gland enlargement was described in only 2 patients [14, 16].

#### Treatment

Desmopressin (DDAVP) was promptly administered in all patients with ADH deficiency [8–10, 13, 14], while glucocorticoid and thyroid hormone replacement therapy was established in patients with anterior pituitary dysfunction [15, 16]. Steroid treatment with methylprednisolone was employed only in a patient exhibiting bilateral optic neuritis [14].

#### Long term outcomes

Most of the patients with ADH deficiency still required treatment at the time of the last reported evaluation, with the longest documented follow-up period being 18 months [12]. Nevertheless, one case reported complete withdrawal of DDAVP therapy and spontaneous full recovery from ADH deficiency [11]. Regarding patients with anterior pituitary dysfunction, progression to complete hypopituitarism was described in a patient with a sellar cystic lesion who declined surgical intervention [15].

#### Pathogenesis

No biopsy data are available; diagnosis relied primarily on clinical and radiological findings. Additionally, autoantibodies were not tested, as noted by some Authors [8, 11]. COVID-19 vaccination was implicated as the causative agent due to its close temporal association with symptoms and the exclusion of common causes of hypophysitis. Three patients had autoimmune predisposition, suggesting a potential autoimmune basis. Authors speculated that hypophysitis may have been triggered by pro-inflammatory state, immunological derangements induced by COVID-19 vaccination, as well as by adjuvants and cross-reactivity mechanisms related to the SARS-CoV-2 spike protein [8–11, 13, 14]. Some Authors hypothesized that vaccine-related hypophysitis could be similar to autoimmune hypophysitis due to immune checkpoint inhibitors (ICI) therapy, except for time of onset (shorter for vaccines) [12, 16].

### Pituitary apoplexy

#### Epidemiology

Post COVID-19 vaccination PA was documented in 6 cases, with an equal sex distribution [17–22]. Mean age was 38 years (range 24–50), being significantly lower in females compared to males (30 vs. 46, *p* = 0.01). Data regarding the vaccine type are available for 5 out of 6 patients. Among these, 3 received a viral vector vaccine (AstraZeneca) while 2 received an mRNA vaccine (Pfizer/BioNTech and Moderna). PA occurred following the 2nd vaccine dose in 3 patients [19, 20, 22] and after the 3rd dose in 1 patient [17]. In one case, initial symptom appeared after the 1st vaccine inoculation (fever for 24 h, headache for 1 month) and intensified after the 2nd dose, accompanied by hyperprolactinemia and amenorrhea prompting MRI evaluation [21]. Nasopharyngeal swabs were used to detect SARS-CoV-2 infection in 3 patients, all yielding negative results [17, 19, 20]. The presence of a pituitary neuroendocrine tumor (PitNET) was generally unknown, except for one patient who had already been diagnosed with a giant PitNET and partial hypopituitarism [19]. Other potential predisposing factors were not highlighted by the Authors.

#### Clinical presentation

PA symptoms occurred shortly after vaccine inoculation, regardless of the vaccine type, with a mean onset delay of 3 days (range 1–5). All patients experienced high-intensity headache, predominantly frontal, characterized by sudden onset and resistance to over-the-counter analgesics. Additionally, headache was variably associated with nausea or vomit, that was present in 2 out of 6 patients [17, 19]; 3 out of 6 patients displayed visual disturbances [17, 19, 22], 2 out of 6 had fever [17, 22] and one out of 6 presented with altered mental status [22]. Two patients presented with hypotension at hospital admission [17, 22]. Regarding hormonal dysfunction, Aliberti et al. documented secondary hypogonadism; ACTH and cortisol were not investigated as the patient promptly underwent dexamethasone treatment due to suspected adrenal insufficiency [17]. No confirmed hypersecretions were reported by other Authors, except for hyperprolactinemia associated with amenorrhea in the study by Roncati et al., which was attributed to the pituitary stalk disconnection effect [21]. ADH deficiency was never reported.

#### Imaging findings

MRI revealed the presence of a giant pitNET in 2 patients [19, 22], macropitNET in 1 patient [17], and a possible micropitNET in 1 patient [18]. Male patients presented with larger pituitary tumors, consistent with the typical diagnostic delay in this gender group [23]. MRI findings showed features of PA, such as signal alterations indicative of recent hemorrhage, mass effect and compression of surrounding structures. Optic chiasm involvement was documented in 4 patients [17, 19, 20, 22], while cavernous sinus extension was observed in 3 patients [17, 20, 22]. Erosion of the back and the floor of sella turcica was noted in 1 patient [17].

#### Treatment

Three patients received immediate stress-dose steroids, primarily hydrocortisone, due to the risk of adrenal insufficiency [18, 19, 22]. Dexamethasone was preferred in 1 patient to reduce edema, and it was coupled with morphine to alleviate headache [17]. Conservative management was chosen in 1 patient who shifted from hydrocortisone to dexamethasone due to persistent headaches and suspicion of hypophysitis, with favourable outcome [20]. Two patients with milder symptoms did not receive any therapy initially, and they shortly experienced resolution [18, 21]. For 3 male patients with large tumors exerting compressive effects on the optic chiasm, surgical approach by transsphenoidal surgery (TSS) was preferred [17, 19, 22].

#### Outcomes

The surgical approach led to headache and visual symptoms improvement [17, 19]. In one case involving a giant pitNET, complete hypopituitarism and post-surgical ADH deficiency were documented, although partial hypopituitarism had already been diagnosed before PA onset [19]. Additionally, Jaggi et al. reported replacement therapy with l-thyroxine and glucocorticoids [22]. Patients managed conservatively experienced symptoms resolution without any functional sequelae [18, 20, 21].

#### Pathogenesis

Various mechanisms have been hypothesized. Some Authors suggest that an exaggerated immunological response triggered by COVID-19 vaccination could lead to PA due to vascular dysfunction [20, 22]. The broad and yet fragile pituitary vascular network is susceptible to endothelial dysfunction and hemorrhage. Zainordin et al. speculated that an enhanced immunological response could have caused hypophysitis and subsequent PA [20]. The hypothesis of hypophysitis was supported by the patient’s young age (24-year-old woman) and the symptoms resolution after dexamethasone treatment [20]. Pinar-Guitiérrez et al. proposed a role for VITT, although thrombocytopenia could not be confirmed in their patient due to the absence of a complete blood count [18]. Notably, none of the reported cases demonstrated thrombocytopenia. Only Aliberti et al. documented increased D-dimer and fibrinogen levels, while platelet count was within the low-normal range [17]. They also conducted immunohistochemistry (IHC) studies on pituitary tissue [17], revealing the expression of SARS-CoV-2 nuclear proteins adjacent to pituitary vessels and the presence of lymphocytic infiltrate. This finding, together with negative SARS-CoV-2 IgM antibody titer, led the Authors to hypothesize that the patient might have had a previous asymptomatic SARS-CoV-2 infection with persistent viral presence in the pituitary tissue. In this context, COVID-19 vaccination likely triggered a robust immunological response, resulting in PA [17].

### Miscellaneous

Other COVID-19 vaccination associated pituitary dysfunctions have been reported.

#### SIADH

SIADH associated transient hyponatremia was described in 5 patients shortly after COVID-19 vaccine administration (within 1–6 days) [24–28]. These patients were generally older (mean age 63.6 years old), although two cases occurred in younger individuals aged 24 and 48 years, respectively. Four cases had received previous mRNA vaccination (2nd and 1st dose), from Pfizer-BioNTech (3/4) and Moderna (1/4), while one patient had received a viral vector vaccination (AstraZeneca). Patients presented with generalized weakness, fatigue and headache, with some experiencing impaired consciousness [25, 26, 28] and nausea/vomiting [26, 28]. All patients exhibited severe hyponatremia (sodium levels 113–118 mEq/l) with low serum osmolality and sodium urinary output > 30 mmol/L. One patient developed status epilepticus [27], and 2 patients had CT findings consistent with brain edema [25, 28]. Management varied: one patient received fluid restriction and oral urea [24], while 4 patients were treated with saline solutions for sodium normalization and symptom control [25–28]. Rapid spontaneous resolution occurred in 3 patients (within 13 h to 7 days) [25, 26, 28]. Authors excluded other potential causes of hyponatremia and SIADH, suggesting that transient SIADH could be triggered by vaccination. They proposed that a cytokine storm, particularly involving pro-inflammatory cytokines like interleukin 6 (IL-6), might stimulate ADH secretion [27]. Furthermore, they noted that SIADH can also result from SARS-CoV-2 infection itself, speculating that the viral spike protein could trigger cytokine release [26]. However, causality in SIADH remains presumed rather than proven.

#### Isolated ACTH deficiency (IAD)

One study reported IAD following immunization with the Pfizer-BioNTech vaccine in a previously healthy 31-year-old man [29]. The patient presented with symptoms and signs of acute adrenal insufficiency, and MRI revealed an atrophic pituitary gland compared to previous normal images. Authors suggested that the vaccine might have initially affected corticotroph cells, leading to adrenal insufficiency, which was precipitated by increased adrenocortical demand induced by vaccination [29].

Another report documented a case of adrenal crisis associated with neuroleptic malignant syndrome following Pfizer vaccination in a 48-year-old man. The patient had no history of hypocortisolism and showed no pituitary abnormalities on MRI. However, he did have a previous history of steroid treatment for glomerulonephritis, which had been discontinued 4 years before [30].

#### Others

COVID-19 vaccination has been linked to precipitating adrenal crisis in 7 patients previously diagnosed with secondary hypocortisolism. These patients, with a mean age of 71 years and mostly men (71%), experienced the crisis within the first 24 h post-vaccination, needing intravenous hydrocortisone treatment [31–34].

Moreover, a recent report suggested the potential association between COVID-19 vaccination and pitNET enlargement in a 60-year-old woman who experienced sudden vision loss 3 days after vaccination [35]. However, establishing causality is challenging, as vision loss can also be the clinical presentation of macropitNETs with suprasellar extension.

## Discussion

COVID-19 vaccination safety and efficacy has been extensively documented [36]. At the same time, endocrine disorders, mainly of autoimmune origin, have been reported, highlighting the need to examine this issue. This review may provide suggestions for the clinical management of pituitary manifestations after COVID-19 vaccination.

**Fig. 1:**
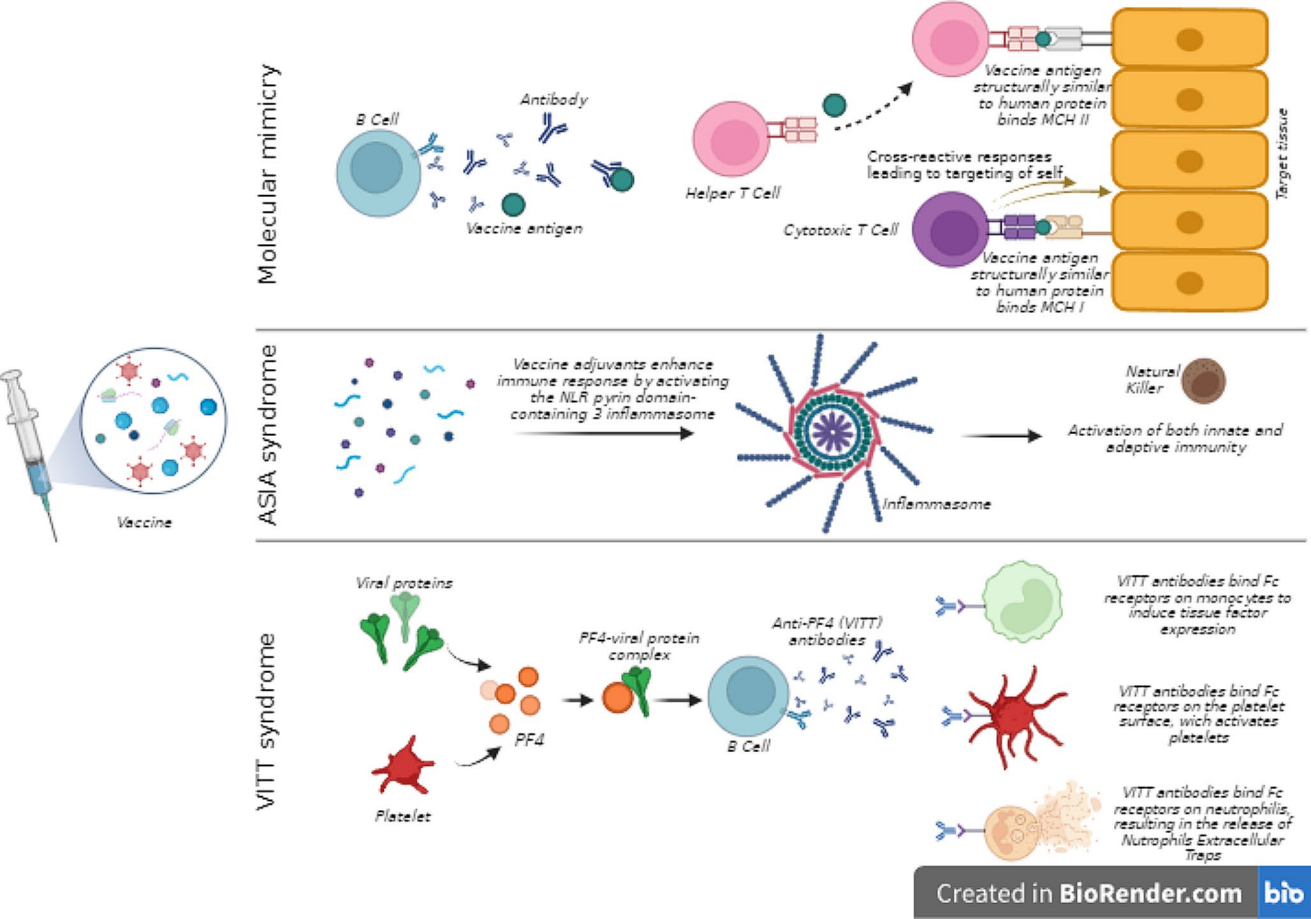
Pathogenetic hypotheses of post COVID-19 vaccination pituitary-related adverse events

The reported cases were vaccinated with mRNA-based vaccine or viral vector vaccine. mRNA-based vaccines (Comirnaty Pfizer/BioNTech and Spikevax, Moderna) consist of the injection of mRNA encoding for the spike protein of SARS-CoV-2, a binding domain that also recognizes human ACE2 receptor, hence triggering an immune response. On the other hand, viral vector-based vaccines (Vaxzevria, Astrazeneca and Jcovden, Janssen) use a weakened adenovirus to deliver SARS-CoV-2 antigens into the host cells and produce an immune response. Both mRNA and adenoviral vector vaccines induce boosted humoral and cell-mediated immune responses through many mechanisms. Molecular mimicry is a type of immune cross-reactivity resulting from the similarity between vaccine components, such as viral S protein, and human proteins. This similarity can lead to an immune response targeting self-antigens (Fig. 1). This mechanism has been suggested to occur in various vaccines, i.e. influenza vaccine, particularly in patients with a genetic predisposition [6]. Vaccine adjuvants, such as oils, mineral salts, lipopolysaccharides, and peptidoglycan, have also been implicated in vaccine-related autoimmune phenomena. Indeed, adjuvants’ role is to enhance immune response by activating the NLR pyrin domain-containing 3 inflammasome, which leads to inflammation and activation of both innate and adaptive immunity [6, 37]. The mRNA itself can serve as an adjuvant. This phenomenon has led to the recognition of the autoimmune/inflammatory syndrome induced by adjuvants (ASIA) within the scientific community [37] (Fig. 1).

According to our literature search, 23 cases of post COVID-19 vaccination pituitary disease have been reported. The majority of findings regarded hypophysitis and PA, but several cases of SIADH and 2 cases of IAD have also been reported. Lastly, although not strictly related to the review topic, documented cases of adrenal crisis in individuals with hypopituitarism following COVID-19 vaccination have been noted. The potential for adrenal crisis was highlighted, with recommendations to increase hydrocortisone only if symptoms like fever, myalgia, or arthralgia develop. Accordingly, a survey conducted by the Pituitary Society showed that most of the clinicians (64%) planned to maintain current glucocorticoid doses with vaccination, escalating only if fever or myalgia/arthralgia developed [38]. Concerns were raised about adrenal crisis occurring with mild or absent fever, especially in older individuals [32]. We believe that caution is warranted in patients with hypocortisolism, but given the rarity of such episodes, further alarmism or overtreatment should be avoided, in agreement with current indications [36].

Hypophysitis was the most commonly reported pituitary disease following COVID-19 vaccination. Hypophysitis usually is a rare condition [39]. The predominant form is lymphocytic hypophysitis, which is thought to be autoimmune-based, while less common forms include granulomatous, xanthomatous, necrotizing and IgG4-related hypophysitis. Additionally, ICI can induce immune-related hypophysitis, particularly monoclonal antibodies against cytotoxic T lymphocyte antigen-4, with an overall incidence of 12% [39]. Similar to the autoimmune form, post-COVID-19-vaccination hypophysitis is more frequent in females, affecting individuals from 16 to 74 years old. Notably, obesity was present in 33% of patients and autoimmunity in another 33%. Hypophysitis was predominantly associated with mRNA vaccines, irrespective of the dose, with onset occurring within a variable period of 3 days to 1 month after vaccination. However, the rarity of these cases does not allow any statistically significant conclusion. Patients with post COVID-19 vaccination hypophysitis mainly presented with hypopituitarism, with the majority developing ADH deficiency, needing desmopressin treatment. Disease was persistent over time, except in one case showing spontaneous recovery [11]. Other cases reported anterior hypopituitarism, mainly central hypocortisolism and hypothyroidism, without recovery. Headache was less frequently reported, and glucocorticoid treatment was used only in one patient with bilateral optic neuritis [14]. Visual disturbances were not observed due to the absence of mass effect on the optic chiasm. Imaging alterations primarily consisted of pituitary stalk thickening and loss of the posterior lobe’s high intensity signal on T1-weighted images rather than pituitary enlargement. In contrast with ICI-related hypophysitis, post-vaccination hypophysitis could manifest earlier, from 3 days to 8 weeks from the injection. Additionally, there is a female predominance, and ADH deficiency is more frequently observed than hypopituitarism. Although some Authors have drawn parallels between these conditions, the different characteristics suggest potentially divergent pathogenetic mechanisms. In all cases the chronological relationship between symptoms onset and vaccine injection along with the exclusion of other possible causes make the causative effect plausible. Few hypotheses were made to establish the possible mechanisms involved in the pathogenesis of post COVID-19 vaccination hypophysitis, although the absence of histological findings does not allow any definitive conclusion. However, Authors agree on a pro-inflammatory state induced by the vaccine components, that activate autoreactive B and T cells in predisposed individuals. This immune reactogenicity could be linked to several mechanisms, mainly S protein molecular mimicry, S protein bond to ACE2 receptor and ASIA syndrome (Fig. 1).

PA has been reported in 6 patients receiving COVID-19 vaccination, regardless of vaccine type or dose. Typically rare, PA presents with sudden severe headache and pituitary hemorrhage. When considering symptomatic cases its incidence is ~ 10% [40]. PA is a medical and surgical emergency due to potential complications like acute adrenal insufficiency and severe visual disturbances [40]. Prompt identification is crucial for better outcomes, emphasizing the need for clinicians to consider PA following COVID-19 vaccination. One of the described cases suggested the possibility of an underlying hypophysitis [20]. However, PA symptom onset was more acute than in hypophysitis, occurring 1 to 5 days post-vaccination. As expected, symptoms always included severe frontal headache, in association with nausea/vomit, visual disturbances, fever, altered mental status and anterior pituitary hormonal dysfunction. Notably, ADH deficiency was never documented. Typical predisposing factors, such as previous surgery, head trauma, hormonal stimulation dynamic tests, angiographic studies, bleeding disorders, and specific drugs [41], were absent, except for COVID-19 vaccination. The younger age of female as compared to male patients may suggest a possible role for estrogens in the development of this adverse event, since no post-menopausal woman had been reported. Based on these data, post COVID-19 vaccination PA shares most of its clinical characteristics with the common form, though its rarity limits epidemiological conclusions.

Vaccines may also lead to thrombosis and bleeding due to VITT [6]. In the setting of COVID-19 vaccination, this mechanism, in combination with pituitary acute stimulation due to inflammatory stress, could further precipitate pituitary ischemia and hemorrhage (Fig. 1). On the other hand, coagulopathy after SARS-CoV-2 infection is well known, presenting as thrombosis at any anatomical site and leading to cerebrovascular accident, myocardial infarction, limb or mesenteric ischemia, deep vein thrombosis and pulmonary embolism [42]. The pathogenesis of this phenomenon involves the binding of SARS-CoV-2 to the ACE2 receptor, which is expressed in the cerebral vascular endothelium. This interaction triggers systemic and vascular inflammation, leading to coagulation cascade activation. Additionally, SARS-CoV-2 binding down-regulates the ACE2 receptor, inducing angiotensin 2 with consequent coagulopathy [42]. SARS-CoV-2 infection is implicated in PA, potentially through COVID-19 related prothrombotic and endothelial systemic illness, particularly in patients with pitNETs [43]. This suggests that the infection may serve as a precipitating risk factor for PA. On this basis, speculation arises regarding post-vaccination PA, considering both an autoimmune (VITT) and a direct SARS-CoV-2 protein effect on the pituitary, especially considering its vascular susceptibility. Immunohistochemistry data confirmed the presence of SARS-CoV-2 nuclear proteins next to pituitary vessels and lymphocyte infiltrate in a recently vaccinated patient with PA, suggesting potential endothelial pituitary vessel infection before vaccination [17]. In this setting, the inflammation caused by COVID-19 vaccination could have triggered PA [17]. However, definitive conclusions about PA pathogenesis in COVID-19 vaccination cannot be drawn since thrombocytopenia was never documented and only one case showed increased D-dimer and fibrinogen levels [17]. In the end, the possibility of PA following COVID-19 vaccination should be taken into account, especially in patients bearing large pituitary tumors.

Transient SIADH forms have been reported in 5 patients after COVID-19 vaccination, mainly in older comorbid individuals with acute onset (1–6 days) and severe hyponatremia. Most patients were recently vaccinated with mRNA vaccines with only one receiving viral vector vaccine. The peculiarity of these cases lies in the rapid resolution (as quick as 13 h). The proposed pathogenesis primarily revolves around the role of IL-6 which stimulates non-osmotic ADH release. SIADH has also been increasingly reported in patients with COVID-19, as well, ascribing this phenomenon to IL-6 and other mechanisms, such as pneumonia-associated ADH release and baroceptor-mediated ADH release in hypovolemic and hypotensive COVID-19 patients [43].

## Conclusions

This review underlines that the pituitary gland can be implicated in adverse events following COVID-19 vaccination. However, unlike thyroid involvement [44, 45], pituitary damage is a much rarer event. The most common manifestations include hypophysitis with ADH deficiency, PA and SIADH. Symptoms typically emerge shortly after vaccine administration. Potential factors contributing to pituitary involvement are the presence of ACE2 receptors in the hypothalamus-pituitary system and a robust inflammatory response in predisposed individuals. Adjuvants may also contribute to boosting the inflammatory response. Furthermore, thrombotic hemorrhagic conditions associated with PA may be linked to ACE2 receptor-related endothelial dysfunction and VITT. The paucity of the reported cases hampers assessing the possible association between specific pituitary adverse outcomes and specific vaccines or comorbidities (Tables 1 and 2). Further studies are necessary to address this issue. However, our findings can aid in diagnostic and treatment decisions for patients presenting with these syndromes. Due to the nonspecific nature of symptom presentation, which can sometimes overlap with more common adverse reactions, pituitary post-vaccination diseases may be underdiagnosed. Nevertheless, given the rarity of these events, safety and efficacy of the currently available COVID-19 vaccines remain very solid and we strongly advocate continuing pursuing vaccination in vulnerable and at-risk individuals.

## Data Availability

No datasets were generated or analysed during the current study.

## Abbreviations

COVID-19: Coronavirus disease 2019
ACE2: Angiotensin converting enzyme 2
VITT: Vaccine-induced thrombotic thrombocytopenia
PF4: Platelet factor 4
PA: Pituitary apoplexy
SIADH: Syndrome of inappropriate antidiuretic hormone secretion
ACTH: Adrenocorticotroph hormone
IAD: Isolated ACTH deficiency
ADH: Anti diuretic hormone
MRI: Magnetic resonance imaging
DDAVP: Desmopressin
ICI: Immune checkpoint inhibitors
PitNET: Pituitary neuroendocrine tumor
TSS: Transsphenoidal surgery
IHC: Immunohistochemistry
IL-6: Interleukin 6
ASIA: Autoimmune/inflammatory syndrome induced by adjuvants
